# Combined intra- and extra-endoscopic techniques for endoscopic intraventricular surgery with a new mini-tubular port

**DOI:** 10.3389/fsurg.2022.933726

**Published:** 2022-08-23

**Authors:** Xi Liu, Yan'kai Qiu, Fan Zhang, Xiaoming Wei, Zhisong Zhou, Feng Zhang, Yiteng Xue, Zhaoru Ma, Xiaosong Wang, Hong Shen, Zhiguo Lin, Huaizhang Shi, Li Liu

**Affiliations:** ^1^ Department of Neurosurgery, The First Affiliated Hospital of Harbin Medical University, Harbin, China; ^2^ Department of Anesthesiology, The First Affiliated Hospital of Harbin Medical University, Harbin, China

**Keywords:** endoscopic intraventricular surgery, tubular port, intra-endoscopic techniques, extra-endoscopic techniques, bleeding

## Abstract

**Objective:**

Intraoperative hemorrhage represents a major risk during endoscopic intraventricular surgery. There are very few publications describing the maintenance of hemostasis during conventional endoscopic intraventricular surgery. Here, we designed a new mini-tubular port to combine intra- and extra-endoscopic techniques for endoscopic intraventricular surgery. With this new methodology, complicated techniques can be performed more efficiently with improved bleeding control.

**Methods:**

The new mini-tubular port consists of an outer sheath and an obturator. The sheath is a thin-walled transparent cylinder that is 0.35 mm thick, 10 mm in diameter, and 90 mm in length. In this report, we describe the use of the mini-tubular port on 36 patients receiving endoscopic intraventricular surgery.

**Results:**

The study enrolled 36 patients, with a median age of 45 years (range: 0–72 years), of which 19 were male and 17 were female. Pure ETV (endoscopic third ventriculostomy) was performed in 20 patients and pure biopsy was performed in 2. ETV and biopsy were performed in five patients, ETV and the removal of cysticerci were performed in five, cyst fenestration was performed in one, ETV and cyst fenestration were performed in two, and ETV and shunt removal were performed in one patient. Two patients received microscopic surgery following endoscopic surgery during the same operation. A total of 17 patients (47%) underwent extra-endoscopic techniques. The median Karnofsky Performance Status (KPS) score of the patients prior to surgery was 50, while the median KPS score of the patients after one month of surgery was 80; these scores were significantly different (*P* < 0.05), as determined by Wilcoxon's test. In total, 27 patients had a KPS score ≥70% and 75% of patients had a favorable prognosis one month after surgery. None of the patients experienced seizure.

**Conclusion:**

The new mini-tubular port can conveniently combine intra- and extra-endoscopic techniques for endoscopic intraventricular surgery. The application of these techniques can efficiently control bleeding during surgery, help improve the confidence of the surgeons involved, and provide a highly efficient approach for performing complicated procedures.

## Introduction

A majority of endoscopic intraventricular surgeries are performed for endoscopic third ventriculostomy (ETV), tumor biopsy, arachnoid cyst fenestration, and colloid cyst resection (1). ETV is the gold standard for obstructive hydrocephalus (2). ETV is being increasingly applied to treat hydrocephalus in subacute patients following bleeding and infection (3).

Conventional endoscopic intraventricular surgery is performed in a fluid medium. Very few special instruments can be used through the working channel of the endoscope; this type of procedure is known as an intra-endoscopic technique (4). During conventional endoscopic intraventricular surgery, an insignificant amount of bleeding can obscure visibility, vascular control can be precarious, and intraoperative hemorrhage is likely the neuroendoscopist's greatest fear during operation, especially when the surgeon has limited experience in endoscopy, as pointed out by Schroeder (1). In minimally invasive endoscopic port surgery, the medium in the surgical field can be changed from water to air, thus leading to a better visualization of hematomas than if a fluid medium was used. During port surgery, regular microsurgical instruments can be used outside of the endoscope; this method is referred to as an extra-endoscopic technique (4). However, very few publications have referred to the management of intraventricular bleeding during conventional endoscopic intraventricular surgery, except for irrigation, small-chamber irrigation, and the dry field technique (DFT) (1, 5). Previously, the endoscopic evacuation of hematomas was carried out by the intra-endoscopic technique; however, in recent years, this type of surgery is performed using the extra-endoscopic technique *via* an endoscopic port.

We gained some experience from techniques of using a dual-channel endoscopic port to eliminate intraventricular hematoma and to implement ETV at the same time (6). Therefore, we designed a new mini-tubular port and applied the intra- and extra-endoscopic techniques to endoscopic intraventricular surgery. This strategy allowed us to perform complicated techniques more efficiently with much greater control over bleeding. Here, we describe the application of our new mini-tubular port in endoscopic intraventricular surgery and demonstrate its effects and advantages.

## Materials and methods

### Description and application of the instrument

**Figure 1:**
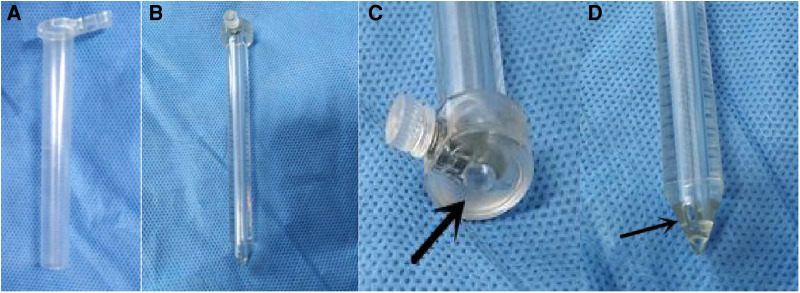
The structure of the new mini-tubular port. (**A**) The outer sheath of the mini-tubular port. (**B**) The obturator of the mini-tubular port. (**C**) The arrow indicates the hollow tube at the proximal end of the obturator. (**D**) The arrow indicates one of the small holes at the distal end of the obturator.

The mini-tubular port consists of an outer sheath and an obturator (Figure 1). The sheath is a thin-walled transparent cylinder that is 0.35 mm thick, 10 mm in diameter, and 90 mm in length. The obturator fits inside the sheath and there is a hollow tube in the center of the obturator. The diameter of the hollow tube is 3.0 mm and accommodates the navigation probe. The distal end of the obturator features two small holes, which connect to the hollow tube. When the port enters the ventricle, the cerebrospinal fluid (CSF) will leak from the hollow tube, thus demonstrating that the port has approached the ventricle in an appropriate trajectory.

### Surgical procedure

The surgical procedure was performed under general anesthesia with the patient in the supine position. A 3- cm linear or curved incision was made at the center of Kocher's point, 1.0 cm anterior to the coronal suture and 2.5 cm lateral to the midline of the head. Then, a 12- mm burr hole was drilled or a 2- cm bone flap was made. The dura mater was coagulated and incised in a cruciate fashion. Then, an 8- mm linear cortical incision was made. A transcortical transventricular puncture was then performed with a common brain puncture needle. When the ventricle was reached, the brain puncture needle was removed. The mini-tubular port was then cannulated according to the trajectory of the brain puncture needle. If the CSF was bloody or turbid, then extra-endoscopic suction would be employed to exchange the CSF with warm Ringer's solution. Extra-endoscopic techniques would be performed to resect cystic lesions from the lateral ventricle or control active bleeding by the application of a conventional slim bipolar, cotton patties, or hemostatic agents such as Surgicel (Ethicon, Inc, Somerville, New Jersey, USA) with a rod rigid endoscope (0 degrees, 4 mm in diameter, and 18 cm in length) (Karl Storz GmbH & Co KG, Tuttlingen, Germany). ETV was performed by intra-endoscopic techniques with the LOTTA Neuroendoscopy system (Karl Storz GmbH & Co KG, Tuttlingen, Germany). An external ventricular drain (EVD) or Ommaya reservoir was then implanted according to the intraoperative conditions.

Initial neurological status was assessed using the KPS at admission. Neurological status was also assessed by determining the KPS score one month postoperatively. Follow-up data were acquired by outpatient review or by telephone interview. Statistical analysis was performed with SAS version 9.4, and Wilcoxon's test was used to determine statistical significance. The level of significance was set at *P *< 0.05.

## Results

Endoscopic intraventricular surgery with the mini-tubular port was performed in 36 patients between March 2015 and December 2021. The median age of these patients was 45 years (range: 0–72 years); 19 males and 17 females. Pure ETV was performed in 20 patients and pure biopsy was performed in 2 patients. ETV and biopsy were performed in five patients, ETV and the removal of cysticerci were performed in another five, cyst fenestration was performed in one, ETV and cyst fenestration were performed in two, and ETV and shunt removal were performed in one patient. Two patients received microscopic surgery following endoscopic surgery during the same operation. Demographic, pathological, and clinical data are presented in Table 1.

**Table 1:** Patient information.

Case	Age (years)	Sex	Diagnosis	Pathology	Surgical procedure	Extra-endoscopic technique	Admission KPS score	1-Month KPS score
1	49	M	Cerebral cysticercosis; hydrocephalus	Cysticercosis	ETV + removal of the cysticerci	+	60	70
2	41	M	Ventriculitis after VP shunt	—	ETV + shunt removal	+	30	0
3	14	M	Third ventricular lesion hydrocephalus	Pineoblastoma	ETV + biopsy	−	30	80
4	68	M	Post-traumatic hydrocephalus	—	ETV	−	30	40
5	48	M	Third ventricular lesion; hydrocephalus	Glioma	Biopsy	−	30	70
6	31	F	Cyst in trigone of the left lateral ventricle	Cyst	Cyst fenestration	+	50	90
7	14	F	Right thalamus lesion; obstructive hydrocephalus	Glioma	ETV + biopsy	−	30	90
8	33	F	Right ventricle and third ventricle lesions; hydrocephalus	Neurocytoma	ETV + biopsy	+	20	80
9	50	F	Basal ganglia hemorrhage breaking into ventricle; hydrocephalus	—	ETV	+	30	50
10	4	M	Suprasellar arachnoid cyst; hydrocephalus	Cyst	ETV + Cyst fenestration	−	60	90
11	2	F	Hydrocephalus after meningitis	—	ETV	−	30	90
12	56	M	Cerebral cysticercosis; hydrocephalus	Cysticercosis	ETV + removal of the cysticerci	−	70	90
13	62	F	Hydrocephalus after subarachnoid hemorrhage	—	ETV	+	40	60
14	38	F	Hydrocephalus after ventricular hemorrhage; Moyamoya disease	—	ETV	+	40	80
15	8	M	Hydrocephalus	—	ETV	−	90	100
16	42	F	Lateral ventricle lesion	Metastasis tumor	Biopsy + microscopic surgery	+	40	100
17	58	M	Cerebral cysticercosis; hydrocephalus	Cysticercosis	ETV	+	70	80
18	35	F	Hydrocephalus	—	ETV	−	70	100
19	53	M	Hydrocephalus; TBM	—	ETV	−	20	70
20	54	M	Cerebral cysticercosis; hydrocephalus	Cysticercosis	ETV + removal of the cysticerci	+	60	80
21	65	F	Hydrocephalus	—	ETV	−	50	60
22	49	F	Hydrocephalus after cerebral hemorrhage	—	ETV	+	40	60
23	35	F	Hydrocephalus	—	ETV	−	60	80
24	53	M	Cerebral cysticercosis; hydrocephalus	Cysticercosis	ETV + removal of the cysticerci	+	60	90
25	64	F	Tumor of pineal region	Differentiating pineal parenchymal cell tumors	ETV	+	60	80
26	38	M	Hydrocephalus	—	ETV	−	60	90
27	23	F	Lateral ventricle lesion; hydrocephalus	Glioma	ETV + biopsy + microscopic surgery	+	70	90
28	53	M	Hydrocephalus	—	ETV	−	60	40
29	57	M	Cerebral lesion	High grade glioma	ETV + biopsy	+	50	80
30	1	M	Hydrocephalus	—	ETV	−	30	50
31	72	F	Cerebral cysticercosis; hydrocephalus	Cysticercosis	ETV + removal of the cysticerci	+	20	50
32	53	M	Tubercular meningitis; hydrocephalus	—	ETV	−	50	80
33	9	M	Hydrocephalus	—	ETV	−	50	90
34	66	F	Suprasellar arachnoid cyst; hydrocephalus	Cyst	ETV + cyst fenestration	+	80	90
35	0	M	Bacterial meningitis; hydrocephalus	—	ETV	−	30	70
36	0	F	Fungal meningitis; hydrocephalus	—	ETV	−	30	70

KPS, Karnofsky Performance Status; ETV, endoscopic third ventriculostomy; VP, ventriculoperitoneal; TBM, tubercular meningitis.

### Application of the extra-endoscopic technique

**Figure 2:**
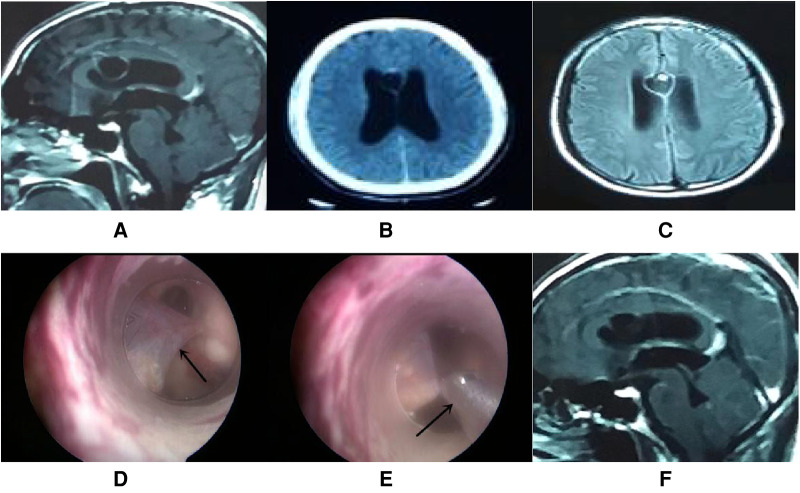
Endoscopic intraventricular surgery was performed to remove a cystic lesion and perform ETV in case 1. (**A–C**) CT and MRI scans showing a cystic lesion with moderate enhancement in the septum pellucidum with a large ventricle. (**D**) Surgical image showing the cystic lesion (arrow point) in the septum pellucidum and connected to the ependyma overlying the caudate. (**E**) Surgical image showing how nasal forceps (arrow point) were used to remove the cystic lesion. (**F**) MRI scan showing that the enhanced cystic lesion had disappeared by day 5 postoperatively.

**Figure 3:**
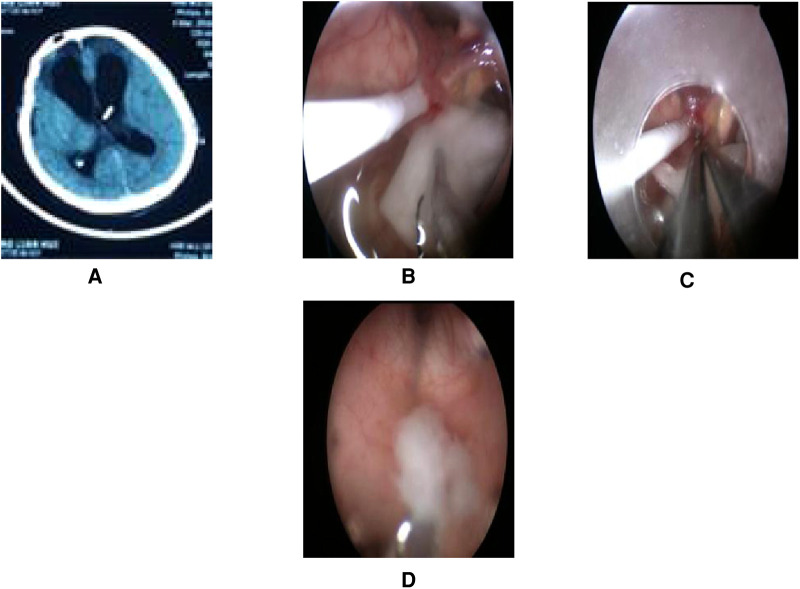
Endoscopic intraventricular surgery was performed to remove the VP shunt tube and to perform ETV in case 2. (**A**) Presurgical CT. (**B**) Surgical image showing intraoperative CSF turbidity, a shunt tube, and choroid plexus adherence. A patty was used to rapidly exchange the CSF with warm Ringer's solution. (**C**) Surgical image showing bipolar coagulation of the choroid plexus vessels with a patty. (**D**) Surgical image showing the removal of purulent secretions at the Cerebral aqueduct of Sylvius by endoscopy.

**Figure 4:**
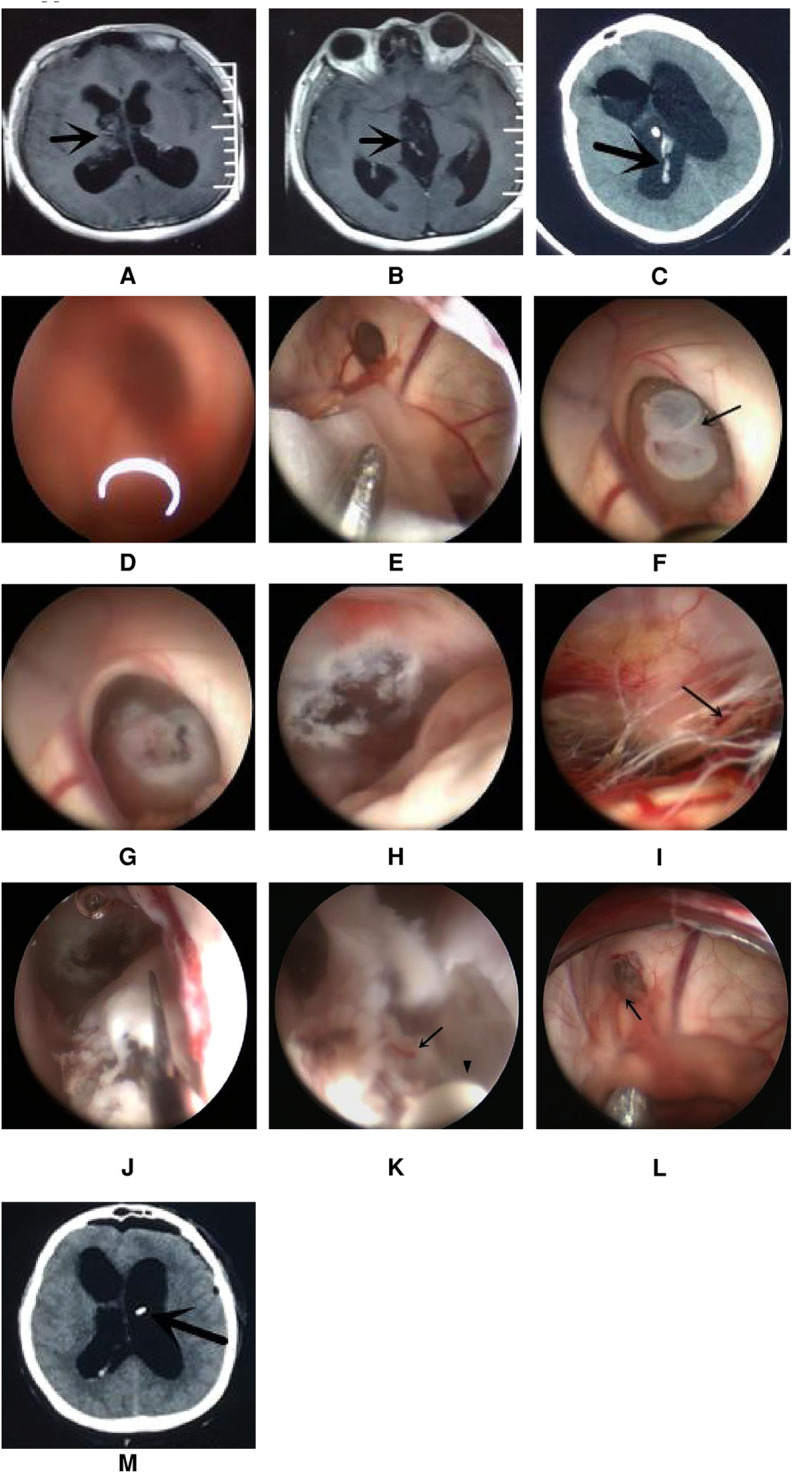
Endoscopic intraventricular surgery was used to perform biopsy and ETV in case 8. (**A,B**) show MRI scans of the lesion in the right lateral ventricle and third ventricle. (**C**) CT scan showing a hemorrhage in the ventricle after EVD. (**D**) Surgical image through the left ventricle showing a blurred image due to a bloody CSF. (**E**) The extra-endoscopic technique was used to clean the image through a slim pledget and suction. (**F**) The lesion was seen to block the foramen of Monro. (**G**) The intra-endoscopic technique was used to coagulate and shrink the tumor. (**H**) Image showing successful ETV with an orifice of more than 5 mm. (**I**) Image showing the prepontine cistern; the arrow shows the abducent nerve. (**J**) The tumor was removed by scissors after ETV. (**K**) Bleeding (arrow point) from a small artery during continuous irrigation at the course of coagulation of concentric circle bipolar (indicated by the arrowhead). (**L**) The source of bleeding (arrow point) was located to a vein close to the foramen of Monro and was stopped by the application of the extra-endoscopic technique. (**M**) CT scan on day 1 after ETV; the arrow shows the Ommaya tube.

**Figure 5:**
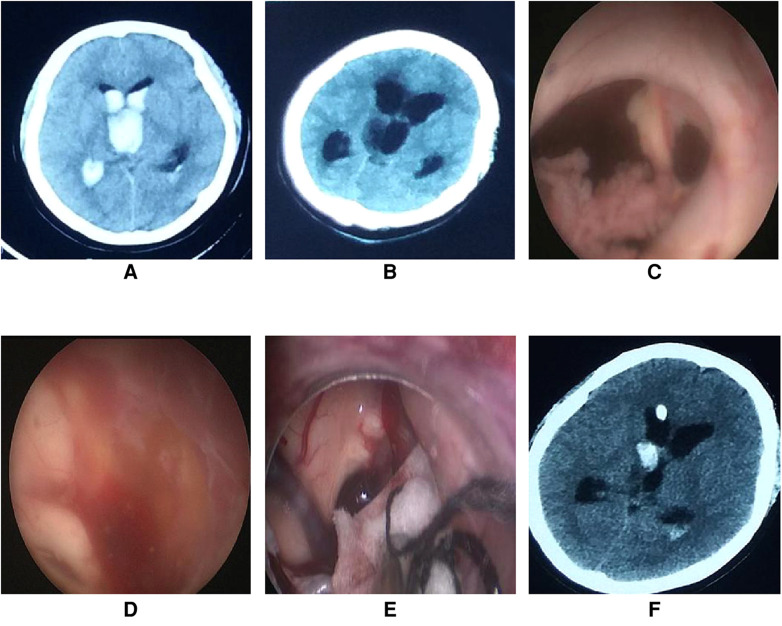
Endoscopic intraventricular surgery was performed to perform ETV in case 14. (**A**) On admission, CT scans revealed a ventricular hemorrhage with hydrocephalus. (**B**) CT scan of hydrocephalus on the third day after extubation of the drainage catheter. (**C**) Connective tissue was found in the foramen of Monro during surgery. (**D**) The third ventricle was full of red-yellow hemosiderin and the bottom of the third ventricle was unrecognizable. (**E**) A patty and slim bipolar was used to stop the bleeding through the extra-endoscopic technique. The source of bleeding was located in the choroid plexus of the lateral ventricle near the foramen of Monro. (**F**) CT scans on the first day after endoscopic surgery showed a small hemorrhage in the right lateral ventricle.

A total of 17 patients (47%) underwent extra-endoscopic techniques. A cystic lesion was removed in case 1 (Figure 2), while the ventricular catheter of a ventriculoperitoneal (VP) shunt was removed in case 2 (Figure 3). The cyst wall was fenestrated in case 6. A bloody and turbid CSF was rapidly cleaned and replaced during surgery involving cases 2, 8, 9, 13, 14, 22, 32, 35, and 36. Cotton patties and Surgicel were used for hemostasis in cases 8 (Figure 4), 14 (Figure 5), 16, 17, 22, and 25.

When complex lesions were encountered, complex operations could be performed in combination with microscopy simultaneously. In case 16, we performed endoscopic biopsy; intraoperative pathological analysis revealed metastases. The tumor did not have a rich blood supply and was resected with a large port (15 mm in diameter) under a microscope. In case 27, ETV and biopsy were performed; intraoperative pathological analysis revealed a low-grade glioma, and tumor resection was performed under microscope by open surgery.

### Case presentation

#### Case 1

This patient was male, 49 years of age, and complained of headache and vomiting for 2 weeks. Computed tomography (CT) and magnetic resonance imaging (MRI) revealed a cystic lesion with moderate enhancement in the septum pellucidum. In addition, the ventricle was larger than normal. The diagnosis was cysticercosis with hydrocephalus. Endoscopic intraventricular surgery was performed and the cystic lesion was removed by the extra-endoscopic technique. ETV was performed with the intra-endoscopic technique (Figure 2). A pathological analysis of the cystic lesion revealed a diagnosis of cysticercosis. Following surgery, the patient received albendazole and recovered well.

#### Case 2

This patient was male and 41 years of age. He was admitted to hospital for ventriculitis following the fitting of a VP shunt. The patient was treated with VP shunt tube removal and ETV by endoscopy. During surgery, we found that the CSF was turbid and that the head of the VP shunt was tightly attached to the choroid plexus. Using the extra-endoscopic technique, a patty was rapidly used to replace the turbid CSF. The head of the shunt tube was removed after bipolar coagulation of the choroid plexus vessels. Finally, ETV was performed successfully. Purulent secretions were removed at the cerebral aqueduct of Sylvius (Figure 3). The video of the surgical procedure is linked to Video 1. Postoperatively, the patient's symptoms improved, although *Acinetobacter baumannii* was cultured from the samples of the CSF. Family members gave up treatment citing financial reasons and the patient was discharged.

#### Case 8

This patient was female and 33 years of age. She underwent open surgery on the right front lobe to resect a ventricular tumor. Pathological analysis revealed a diagnosis of the central neurocytoma. She suffered from hydrocephalus and the residual of the tumor after the operation. A VP shunt was not suitable for this patient because leukocyte and protein levels were high in the CSF. Due to acute tonsillar herniation arising from the hydrocephalus, the patient received external ventricular drainage (EVD). CT scans showed a small hemorrhage in the ventricle. She received ETV and Ommaya reservoir implantation through the left ventricle on the third day after EVD. The bloody CSF resulted in blurred images through the lens. Thus, the extra-endoscopic technique was used to flush out the bloody CSF through a slim pledget. The tumor was found at the foramen of Monro and was coagulated simultaneously *via* the intra-endoscopic technique. ETV was performed after shrinking the tumor. We tried to aggressively resect the tumor following ETV. Slight bleeding occurred during the process of resection; this was stopped by repeated electrocoagulation using a concentric circle bipolar under the intra-endoscopic technique, which took approximately 3 min to perform. After further exploration, severe venous bleeding occurred behind the lens and could not be stopped by copious irrigation, thus blurring the surgical field. Fortunately, the bleeding was stopped by the extra-endoscopic technique with the aid of cotton patties. The bleeding site was located in a vein close to the foramen of Monro. Because the patient's relatives rejected our request to remove the residual tumor by open surgery, the tumor was left *in situ* and an Ommaya reservoir was implanted (Figure 4). The patient recovered after ETV and received radiotherapy after discharge; the symptoms of hydrocephalus subsequently disappeared.

#### Case 14

This case was a 38-year-old woman who was admitted to hospital for ventricular hemorrhage, obstructive hydrocephalus, and Moyamoya disease. Bilateral emergency EVD was performed after admission. Urokinase injection *via* an EVD catheter was performed twice a day for resolving the hematoma. On the eighth day after EVD, a CT scan showed that the hematoma in the ventricle disappeared. After clamping the drainage catheter for 2 days, the CT scan showed no hydrocephalus, following which the drainage catheters were extubated. On the third day after extubation of the drainage catheter, the patient developed sluggishness, and a CT scan showed the hydrocephalus. A VP shunt was not suitable because of the characteristics of the CSF. ETV was found to be better than EVD after consultation with the relatives of the patient, and thus, ETV was performed.

Connective tissue was found in the foramen of Monro during the operation. Exploration revealed that the third ventricle was full of red-yellow hemosiderin and the bottom of the third ventricle was unrecognizable. Active bleeding occurred behind the endoscope. Because the bleeding could not be controlled by irrigation, the extra-endoscopic technique was used to perform hemostasis. A patty and bipolar were applied and successfully stopped the bleeding. As the bottom of the third ventricle could not be identified, ETV was terminated and EVD was performed (Figure 5). The video of the surgical procedure is linked to Video 2. Postoperatively, the hydrocephalus symptoms were relieved and the KPS score was 80 points when assessed one month after surgery. As yet, a VP shunt has not been fitted.

### Treatment outcomes and complications

All 36 patients were treated successfully. The median KPS score of the patients before surgery was 50 and the median KPS score of the patients after one month of surgery was 80. Wilcoxon's test revealed that the difference in KPS scores before surgery and one month of surgery was statistically significant (*P *< 0.05). There were 27 patients whose KPS scores were ≥70%, and 75% of patients had a favorable prognosis after one month of surgery.

One patient (case 2) was discharged with an intracranial infection and gave up treatment; this patient died one month after surgery. Intraoperative hemorrhage was observed in case 14; although ETV was not employed, the symptom of hydrocephalus in this case was successfully resolved. Transient subcutaneous fluid accumulated as a result of CSF leakage in one patient, who recovered after relevant treatment. None of the patients suffered postoperative seizure.

## Discussion

Conventional endoscopic intraventricular surgery is increasingly being used for ETV, biopsy, and the resection of colloid cysts. However, in recent times, ETV is also being increasingly used to treat hydrocephalus in the subacute stage after bleeding and infection; these particular cases experience greater levels of bleeding than regular cases (3).

In a previous study, Li et al. reported hemorrhage in 75 (59.9%) of the 126 patients treated in their study of endoscopic intraventricular surgery; these authors divided intraoperative intraventricular hemorrhage into three types (7). In the conventional endoscopic intraventricular surgery, the medium is the liquid; even insignificant bleeding can obscure visibility, for which only the special bipolar and irrigation can be used (1, 5). For little arterial bleeding, continuous irrigation should be proceeded until the bleeding site is found, and then the bleeding artery is ceased by special bipolar electrocoagulation. For venous bleeding and large arterial bleeding, nevertheless, there is no effective method of hemostasis because the bleeding site cannot be identified clearly after irrigation. As Schroeder said in his paper, it may be difficult to control bleeding from free-floating vessels, e.g., septal vein injury (1), by irrigation.

Vascular control during endoscopic intraventricular operation is precarious, and this is likely the neuroendoscopist's greatest fear. In 2014 and 2018, the small-chamber irrigation technique (SCIT) and the DFT were introduced, respectively, by Schroeder (1, 5) to manage intraoperative hemorrhage during ventriculoscopic surgery. The SCIT is equivalent to creating a port that can provide a small space around the bleeding vessel, while the DFT technique is equivalent to an air environment created by a tubular port during the performance of extra-endoscopic techniques. In theory, the extra-endoscopic technique by mini-port is equivalent to a combination of the SCIT and DFT (1, 5). In addition, when performed with a mini-port, the extra-endoscopic technique can allow the use of cotton patties and microsurgical instruments during surgery, such as a slim bipolar; these advantages can improve the efficiency of complicated procedures. The mini-tubular port can provide prearranged plans for controlling bleeding and improving the confidence of the surgeon. Endoscopic hematoma evacuation has been transferred from the intra-endoscopic technique to the extra-endoscopic technique by port (1). The endoscopic intraventricular surgery by the mini-port will be a promising option for combining intra- and extra-endoscopic techniques for endoscopic intraventricular surgery.

### Advantages of the mini-tubular port

Initially, the sheath used for endoscopic intraventricular surgery was metal; however, this practice has been abandoned due to several drawbacks. At present, most surgeons use a transparent peel-away sheath (8, 9). The routine peel-away sheath is 7 mm in diameter; this is approximately 2 French larger than the diameter of the endoscope. We independently designed a new mini-tubular port with a diameter of 10 mm. The length of the obturator is approximately 90 mm. There is a hollow tube in the center of the obturator. The diameter of the tube is 3.0 mm, thus accommodating the navigation probe (Figure 1). The distal end of the obturator features two small holes that connect to the hollow tube. When the port enters the ventricle, the CSF will leak from the hollow tube, thus demonstrating that the trajectory to the ventricle is correct.

### Safety aspects of the mini-tubular port

The MINOP Invent Trocar (FH620R) is 8.3 mm in diameter (Aesculap, Inc); the diameter of this trocar, in combination with a transparent peel-away sheath, could reach up to 8.6 mm (10). Cohengadol et al. reported that the diameter of the mini-tubular port used in their study was 12 mm (11). The mini-tubular port used in the present study is 10 mm in diameter and therefore 1.4 mm larger than the MINOP Invent Trocar. The port redistributes pressure equally across the surrounding tissue and produces less direct cutting and/or tearing trauma to the underlying brain tissue (12, 13). Therefore, in theory, our new mini-tubular port does not cause additional damage to the brain tissue and avoids serious complications. In the present study, none of the 36 patients who underwent endoscopic intraventricular surgery with the mini-tubular port suffered serious complications such as epilepsy; these findings were consistent with those of a previous study (11).

In theory, a 10- mm port might establish a pathway from the ventricle to the convexity subarachnoid space after surgery; this may relieve the symptoms of hydrocephalus in line with a Torkildsen shunt (14, 15). This might be the reason why postoperative hydrocephalus symptoms were alleviated in case 14, even though ETV was not performed intraoperatively and a VP shunt was not placed after 2 years of follow-up.

But at the same time, the 10- mm port could increase the risk of a local unilateral subdural collection. We will demonstrate the effect of a larger pathway from the ventricle to the convexity subarachnoid space in a large number of cases in the future.

### Application notes

CSF leakage is the second most common complication after endoscopic intraventricular surgery, ranging from 1.7% to 5.2% (16–18). In order to prevent the occurrence of CSF leakage after surgery, we used a bone flap; we also used an artificial dura mater to create water-tight sutures. In addition, the curved flap incision allowed the mini-tubular port of 10 mm to be rapidly replaced by a larger port to stop bleeding in accordance with the bimanual procedure by microscopy or endoscopy according to intraoperative conditions (6, 19).

The diameter of our modified mini-port is 10 mm. In theory, the modified mini-port will let more potential damage to the brain tissue than the 7- mm peel-away sheath in conventional endoscopic intraventricular surgery. Thus, the indication of these techniques with the new 10mm mini port is especially suitable for endoscopic intraventricular surgery such as intraventricular lesion biopsy/removal and ETV for patients in the subacute after bleeding and infection, in which the risk of bleeding is high and need more complicated procedures.

### Pitfalls of the device

The diameter of the mini-tubular port is 10 mm; this may restrict the ability to maneuver instruments. When cases involve more complicated procedures or significant arterial bleeding, it is necessary to use bimanual procedures; the mini-port will not provide sufficient space for bimanual procedures. The mini-port can be replaced by a larger port (up to a diameter of 15 mm) or retractors used in open surgery. Two cases were converted to microscopic tumor resection after neuroendoscopic biopsy.

### Study limitations

The study included only 36 patients. Besides KPS, we feel that some other indicators such as surgery time and cortical damage will more precisely demonstrate the advantage of the modified mini-tubular fort. We will significantly enlarge our sample size to ascertain the advantage offered by the modified mini-tubular port in the future by other indicators, including the two mentioned above.

## Conclusion

The new mini-tubular port described herein can conveniently combine intra- and extra-endoscopic techniques for endoscopic intraventricular surgery. The application of these two techniques can efficiently control bleeding during surgery and improve the confidence of surgeons, especially when the surgeon has limited experience in endoscopy. Furthermore, this technique can improve the efficiency of complicated procedures. In addition, a 10- mm port can establish a pathway from the ventricle to the convexity subarachnoid space after surgery, thus relieving the symptoms of hydrocephalus.

## Data Availability

The original contributions presented in the study are included in the article/Supplementary Material, further inquiries can be directed to the corresponding authors.

## References

[1] Manwaring JC El Damaty A Baldauf J The small-chamber irrigation technique (SCIT): a simple maneuver for managing intraoperative hemorrhage during endoscopic intraventricular surgery. Neurosurg. (2014) 3:375–9. 10.1227/NEU.0000000000000406 24867199

[2] Di Vincenzo J Keiner D Gaab MR Endoscopic third ventriculostomy: preoperative considerations and intraoperative strategy based on 300 procedures. J Neurol Surg A Cent Eur Neurosurg. (2014) 75(1):20–30. 10.1055/s-0032-1328953 23733264

[3] Chrastina J Novák Z Zeman T The results of neuroendoscopic surgery in patients with posttraumatic and posthemorrhagic hydrocephalus. World Neurosurg. (2018) 113:e113–21. 10.1016/j.wneu.2018.01.186 29408347

[4] Cai R Di X. Combined intra- and extra-endoscopic techniques for aggressive resection of subependymal giant cell astrocytomas. World Neurosurg. (2010) 73(6):713–8. 10.1016/j.wneu.2010.02.068 20934162

[5] Oertel J Linsler S Csokonay A Management of severe intraoperative hemorrhage during intraventricular neuroendoscopic procedures: the dry field technique. J Neurosurg. (2018) 131(3):931–5. 10.3171/2018.4.JNS172537 30239311

[6] Liu L Liu X Zhang F Dual-channel minimally invasive endoscopic port for evacuation of deep-seated spontaneous intracerebral hemorrhage with obstructive hydrocephalus. World Neurosurg. (2016) 91:452–9. 10.1016/j.wneu.2016.04.066 27132183

[7] Li C Zong X Wang X Intraoperative hemorrhage in ventriculoscopic surgery: experience of a single Chinese neurosurgery center. World Neurosurg. (2016) 88:548–51. 10.1016/j.wneu.2015.10.041 26514635

[8] Marx S Schroeder H. Benefits of endoscopic sheath in intraventricular neuroendoscopy: technical note. J Neurol Surg A Cent Eur Neurosurg. (2021) 82(06):594–8. 10.1055/s-0040-1701618 34010982

[9] Abdullah SH. New transparent peel-away sheath with neuroendoscopic orientation markers: technical note. J Neurosurg. (2007) 107(6):1244–7. 10.3171/JNS-07/12/1244 18077967

[10] Shim KW Park EK Kim DS Neuroendoscopy: current and future perspectives. J Korean Neurosurg Soc. (2017) 60(3):322–6. 10.3340/jkns.2017.0202.006 28490159 PMC5426450

[11] Cohengadol AA. Minitubular transcortical microsurgical approach for gross total resection of third ventricular colloid cysts: technique and assessment. World Neurosurg. (2013) 79(1):207.e7–e10. 10.1016/j.wneu.2011.03.045 22120401

[12] Bander ED Jones SH Kovanlikaya I Utility of tubular retractors to minimize surgical brain injury in the removal of deep intraparenchymal lesions: a quantitative analysis of FLAIR hyperintensity and apparent diffusion coefficient maps. J Neurosurg. (2016) 124(4):1053–60. 10.3171/2015.4.JNS142576 26430838

[13] Almenawer SA Crevier L Murty N Minimal access to deep intracranial lesions using a serial dilatation technique: case-series and review of brain tubular retractor systems. Neurosurg Rev. (2013) 36(2):321–9. 10.1007/s10143-012-0442-x 23224780

[14] Morota N Ihara S Araki T. Torkildsen shunt: re-evaluation of the historical procedure. Childs Nerv Syst. (2010) 26(12):1705–10. 10.1007/s00381-010-1182-2 20502902

[15] Teegala R. Trans aqueductal, third ventricle – cervical subarachnoid stenting: an adjuvant cerebro spinal fluid diversion procedure in midline posterior fossa tumors with hydrocephalus: the technical note and case series. Asian J Neurosurg. (2016) 11(3):268–72. 10.4103/1793-5482.145369 27366254 PMC4849296

[16] Bouras T Sgouros S. Complications of endoscopic third ventriculostomy. World Neurosurg. (2013) 79(2 Suppl):S22.e9–e 12. 10.1016/j.wneu.2012.02.014 22381818

[17] Yadav YR Parihar VS Ratre S Avoiding complications in endoscopic third ventriculostomy. J Neurol Surg A Cent Eur Neurosurg. (2015) 76(6):483–94. 10.1055/s-0035-1551828 26140421

[18] Kawsar KA Haque MR Chowdhury FH. Avoidance and management of perioperative complications of endoscopic third ventriculostomy: the Dhaka experience. J Neurosurg. (2015) 123(6):1414–9. 10.3171/2014.11.JNS14395 26024001

[19] Hong CS Prevedello DM Elder JB. Comparison of endoscope- versus microscope-assisted resection of deep-seated intracranial lesions using a minimally invasive port retractor system. J Neurosurg. (2016) 124(3):799–810. 10.3171/2015.1.JNS141113 26315005

